# Evaluating the implementation of a multi-level mHealth study to improve hydroxyurea utilization in sickle cell disease

**DOI:** 10.3389/frhs.2022.1024541

**Published:** 2023-01-20

**Authors:** J. S Hankins, M. B Potter, M. E Fernandez, C Melvin, L DiMartino, S. R Jacobs, H. B Bosworth, A. A King, J Simon, J. A Glassberg, A Kutlar, V. R Gordeuk, N Shah, A. A Baumann, L. M Klesges, the Sickle Cell Disease Implementation Consortium

**Affiliations:** ^1^ Department of Hematology, St. Jude Children's Research Hospital, Memphis, TN, United States; ^2^ Department of Family and Community Medicine, University of California San Francisco School of Medicine, San Francisco, CA, United States; ^3^ Clinical, Family, and Community Medicine, University of California San Francisco, CA, United States; ^4^ Health Promotion and Behavioral Sciences, University of Texas Health Science Center, School of Public Health at Houston, Houston, TX, United States; ^5^ The Medical University of South Carolina, Charleston, SC, United States; ^6^ RTI International, Research triangle park, NC, United States; ^7^ Department of Population Health Studies, Duke University, Durham, NC, United States; ^8^ Center of Innovation to Accelerate Discovery and Practice Transformation (ADAPT), Durham Veterans Affairs Medical Center, Durham, NC, United States; ^9^ Department of Pediatrics, Washington University, Saint louis, MO, United States; ^10^ Department of Emergency Medicine, Ichan School of Medicine at Mount Sinai, New York, NY, United States; ^11^ Augusta University, Augusta, GA, United States; ^12^ Department of Medicine, the University of Illinois at Chicago, Chicago, IL, United States; ^13^ Department of Pediatric Hematology and Oncology, Duke University, Durham, NC, United States; ^14^ Division of Public Health Sciences, Department of Surgery, Washington University, Saint louis, MO, United States

**Keywords:** sickle cell anaemia (SCA), frameworks, process development and design, hydroxycarbamide, adherence, team science

## Abstract

**Background:**

Sickle Cell Disease (SCD) is a progressive genetic disease that causes organ damage and reduces longevity. Hydroxyurea is an underutilized evidence-based medication that reduces complications and improves survival in SCD. In a multi-site clinical trial, part of the NIH-funded Sickle Cell Disease Implementation Consortium (SCDIC), we evaluate the implementation of a multi-level and multi-component mobile health (mHealth) patient and provider intervention to target the determinants and context of low hydroxyurea use. Given the complexity of the intervention and contextual variability in its implementation, we combined different behavioral and implementation theories, models, and frameworks to facilitate the evaluation of the intervention implementation. In this report, we describe engagement with stakeholders, planning of the implementation process, and final analytical plan to evaluate the implementation outcomes.

**Methods:**

During 19 meetings, a 16-member multidisciplinary SCDIC implementation team created, conceived, and implemented a project that utilized Intervention Mapping to guide designing an intervention and its evaluation plan. The process included five steps: (1) needs assessment of low hydroxyurea utilization, (2) conceptual framework development, (3) intervention design process, (4) selection of models and frameworks, and (5) designing evaluation of the intervention implementation.

**Results:**

Behavioral theories guided the needs assessment and the design of the multi-level mHealth intervention. In designing the evaluation approach, we combined two implementation frameworks to best account for the contextual complexity at the organizational, provider, and patient levels: (1) the Consolidated Framework for Implementation Research (CFIR) that details barriers and facilitators to implementing the mHealth intervention at multiple levels (users, organization, intervention characteristics, broader community), and (2) the Technology Acceptance Model (TAM), a conceptual model specific for explaining the intent to use new information technology (including mHealth). The Reach Effectiveness Adoption Implementation and Maintenance (RE-AIM) framework was used to measure the outcomes.

**Discussion:**

Our research project can serve as a case study of a potential approach to combining different models/frameworks to help organize and plan the evaluation of interventions to increase medication adherence. The description of our process may serve as a blueprint for future studies developing and testing new strategies to foster evidence-based treatments for individuals living with SCD.

## Introduction

Sickle cell disease (SCD) is a devastating genetic disease where progressive organ damage leads to premature death (1). Only a few evidence-based treatments exist for SCD, such as the disease-modifying agent hydroxyurea, and all are vastly underutilized. Uptake of disease-modifying therapies by patients with SCD is impacted by socio-economic barriers that create health inequities for this population (2–4). To accelerate the equitable translation of evidence-based treatments into clinical care for individuals with SCD, the Sickle Cell Disease Implementation Consortium (SCDIC) was created in 2016 (5). The SCDIC is a cooperative research program funded by the NHLBI and composed of eight academic and clinical sites and one data coordinating center. SCDIC members include clinicians, epidemiologists, implementation scientists, government representatives, behavioral scientists, and patient partners. A primary goal of the SCDIC is to develop and test the effectiveness of interventions aimed at increasing the uptake of evidence-based therapies for SCD while seeking input from key stakeholders during the entire implementation strategy development process (5).

As the first FDA-approved evidence-based drug for SCD, daily oral hydroxyurea is a medication recommended by guidelines (6) and with robust evidence for reducing acute and chronic disease complications, lowering acute-care utilization, and improving survival among individuals with SCD (7–11). In the U.S., <50% of patients with SCD appropriately utilize hydroxyurea (12–15), severely limiting its population impact. SCDIC consortium members thus chose to focus on strategies to increase hydroxyurea uptake. The consortia members and stakeholder partners worked together to develop a multi-level/multi-component mobile health (mHealth) intervention to increase provider prescribing practices and patient medication adherence to hydroxyurea. The implementation of this novel mHealth intervention is currently being tested in a multicenter study (16, 17).

During the development of the multi-level/multi-component mHealth intervention, a major challenge within the SCDIC was to construct an evaluation approach to identify how each contextual level (i.e., at the patient, provider, and clinical setting) and intervention component (i.e., the different mHealth features) could influence implementation and effectiveness outcomes, both as individual factors and interactively. For instance, patients, providers, and institutional characteristics can influence the implementation and distal effectiveness outcomes. Still, implementation outcomes can also interact across multiple levels (e.g., provider adoption might impact patient reach), modifying the effectiveness outcomes. To address this challenge, we used the intervention mapping methodology (18), which allows for strong collaboration among researchers and patient partners to develop the multi-level/multi-component mHealth intervention and to guide the design of the evaluation plan.

In this paper, we will report the process development of an intervention evaluation plan that considers *what* and *how* contextual factors influence implementation outcomes while anticipating possible interrelationships (e.g., synergy) among the intervention components. This paper will discuss different theories, models, and frameworks and how they are optimally combined to evaluate the implementation of a multi-level/multi-component mHealth intervention to improve hydroxyurea utilization in SCD (clinicaltrials.gov NCT03344900) (17). We describe the rationale, process, and application of the implementation process and the engagement with stakeholders to develop the analytical plan of the intervention and define implementation outcomes. The description of our design process may serve as a blueprint for future studies developing and testing new strategies to foster the use of evidence-based treatments for individuals living with SCD.

## Methods

This descriptive narrative of the process selects and blends theories, models, and frameworks to design the clinical trial evaluation that tests a new multi-level/multi-component mHealth intervention to improve hydroxyurea adherence among patients with SCD (17).

### Settings and population

This multi-center study included seven SCDIC clinical sites, all academic institutions in urban, suburban, and rural areas, with some degree of organizational and population variability. All sites have experienced SCD providers (adult and pediatric hematologists and advanced care practitioners, all trained in SCD management) and trainees (e.g., residents and fellows). The prevalence of eligible patient participants (i.e., patients with SCD treated with hydroxyurea who were not receiving monthly blood transfusions and not using mobile apps to improve adherence) ranged from 40 to 60% of the patient population at each site. Among eligible participants, approximately 70% were covered under government health plans (primarily Medicaid). Most patients were aged >25 years, although, in two sites, about half of eligible patients were adolescents. Among eligible patients, approximately 70% had a severe SCD genotype (HbSS and HbS*β*^0^-thalassemia).

### Research team

Our SCDIC team comprises 16 members, including implementation scientists, hematologists, health science researchers, behavioral scientists, research coordinators, biostatisticians, patient partners, clinicians, and epidemiologists who collaborated on the study throughout 19 meetings from December 2017 to October 2019. In addition to the research team, the SCDIC steering committee (including principal site investigators, NHLBI representatives, patient partners, data coordinating center staff, and study coordinators) and the SCDIC Implementation Research Committee (composed of implementation scientists) offered an additional layer of scientific input, suggested changes to the study, and voted to approve the study in its final form.

### Summary of activities

Our activities began by providing level-setting knowledge about SCD and dissemination and implementation research to all members of the SCDIC. As the research team was diverse and background knowledge was often non-overlapping, much of the initial work centered on the generation of a shared vision, i.e., establishing agreement on the goals of the program and the process whereby a research question was developed and addressed. The design of the intervention components followed a comprehensive needs assessment phase examining the barriers and enablers to hydroxyurea utilization in the SCD population at multiple levels (Activity 1). Next, a conceptual model evolved using Intervention Mapping (Activity 2) to guide the development of the interventions and the planning of the implementation strategies (Activity 3), the selection of the models and frameworks (Activity 4), and the evaluation plan (Activity 5). We used an inclusive consensus approach throughout all activity phases and defined and selected priorities, designed interventions, and measured outcomes. Our study is fully accrued and is on track to complete data analysis by March 2023. Next, we will present the details of each activity.

### Activity 1: Needs assessment of hydroxyurea utilization

Our needs assessment focused on care redesign to improve hydroxyurea uptake and considered intervention targets, modalities, and strategy mechanisms. Barriers and enablers to hydroxyurea utilization in the SCD population were conducted through literature synthesis (19–22), population-level claims analysis (13, 23), patient and provider surveys (24, 25), semi-structured interviews, and focus groups (16, 26). To synthesize and organize the needs assessment findings at the patient level, the Health Belief Model (HBM) (27) was used, and the Social Cognitive Theory (SCT) was used as the behavioral change model (28). The HBM has been broadly used to evaluate the acceptance of health services among people with SCD, including attendance to clinic visits (29), sepsis (30), and stroke screening (31). The constructs within the HBM align well with the disease characteristics and demographics (e.g., intermittent acute exacerbations align with perceived susceptibility), therefore, was selected for this project. In alignment with our behavioral models, our needs assessment findings identified the main determinants of poor hydroxyurea adherence among patients with SCD: perceived high susceptibility, high disease severity, low motivation to take medications, memory deficit (leading to poor habituation), low understanding of hydroxyurea benefit (i.e., medication knowledge), and low self-efficacy with taking hydroxyurea (16, 26). Among providers of patients with SCD, the main determinants of low rates of prescribing hydroxyurea are: limited knowledge of the drug, lack knowledge of the national care guidelines, and low self-efficacy in dosing hydroxyurea (24, 32). Following this formative evaluation, our team identified two priority target levels for intervention: 1) patient hydroxyurea adherence and 2) provider prescribing of hydroxyurea.

### Activity 2: Development of the conceptual model

Informed by the needs assessment, we developed a comprehensive conceptual model centered on Intervention Mapping. To organize the components of the intervention at both the patient and the provider levels, we used Intervention Mapping to identify specific methods and practical applications to create change in determinants, performance objectives, and behavioral change outcomes. Intervention Mapping is a protocol based on using theory and evidence for developing effective behavior change interventions (18). A primary aim of the consortia was to develop a research protocol that included a consensus framework. Drawing on the team's expertise and experience in using different implementation frameworks, we selected frameworks based on overarching aims (33). For instance, to understand what influences implementation, we selected a determinant framework, and to evaluate the implementation we chose an evaluation framework. We thus combined different models and frameworks to 1) identify determinants of poor hydroxyurea utilization at multiple levels, 2) guide the design of the interventions and strategies to be used at different intervention levels, and 3) plan the implementation evaluation. This comprehensive process allowed us to identify the salient targets to increase the use of the evidence-based treatment for SCD, hydroxyurea, while identifying and prioritizing important influencers of the implementation at the contextual level.

### Activity 3: Intervention design process

SCDIC investigators noted the widespread use of technology among patients with SCD (>90% own smartphones, 91% use them regularly for communication, and 87% rate the highest possible comfort levels (34, 35) and the desire of providers to receive SCD guideline information in a mobile platform (36). The research team arrived at the consensus that to best “package” all necessary functions of the intervention and deliver it to the two targets (patients and providers), mHealth was the ideal conduit. mHealth intervention refers to the use of mobile technology for medical and public health practices (37) and here it serves as the channel for effecting behavioral modifications.

**Figure 1:**
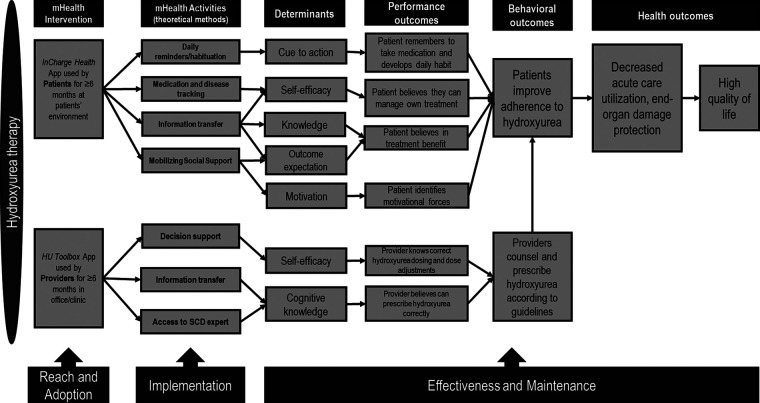
Multi-level/multi-component intervention mapping and implementation outcomes. The intervention components (mHealth activities) address the determinants of hydroxyurea utilization at the patient and provider levels. Performance outcomes depict the actions taken by the targets of the intervention. Provider-appropriate prescribing influences patients’ hydroxyurea adherence, promoting increased hydroxyurea utilization, reduced organ damage, and improved quality of life.

**Figure 2:**
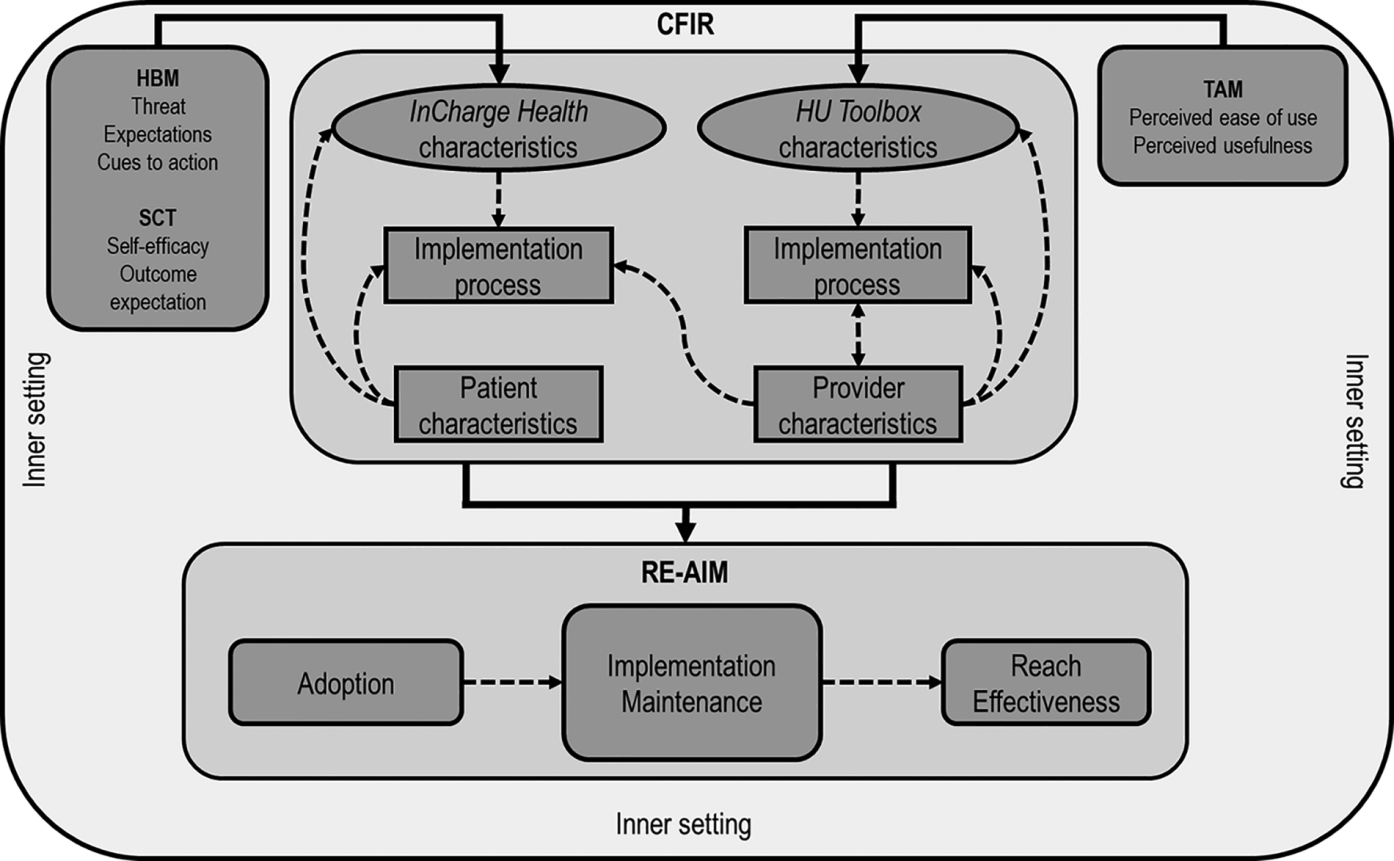
Interrelation of theories, models, and frameworks. The Health Belief Model (HBM) and Social Cognitive Theory (SCT) informed the design of the *InCharge Health* app. Technology Acceptance Model (TAM) informed the creation of the *HU Toolbox.* The Consolidated Framework for Implementation Research (CFIR) constructs will inform the evaluation of the multiple-level influential factors, including the inner setting (organization), the users (patients and providers), intervention characteristics (*InCharge Health* and *HU Toolbox* apps), and app implementation process. The Reach Effectiveness Adoption implementation and Maintenance (RE-AIM) framework domains will be used to evaluate the implementation process. Dashed arrows represent the possible influential effects across CFIR constructs and RE-AIM domains.

Using user-centered design principles, we co-created (with patients and providers) a two-level intervention: (1) *InCharge Health* app, which incorporated features that acted on the modifiable determinants of patients' poor hydroxyurea adherence (e.g., low cue to action, low self-efficacy in taking medication) (Figure 1), and (2) *HU Toolbox app* for providers, which incorporated features that acted on modifiable determinants of clinician failure to prescribe hydroxyurea (e.g., low disease knowledge, low self-efficacy in prescribing hydroxyurea) (Figure 1). User-centered design is an approach to designing and developing products that grounds its process on the information about the people who will ultimately use the products to improve usability and user experience (38, 39). Patients used *InCharge Health* within their own lived environment, while providers used *HU Toolbox* in the clinic/office. Because in clinical practice, providers prescribe and counsel patients on the benefits of hydroxyurea during regular visits, providers were both the actors and targets of our multi-level intervention (Figure 1) (40).

A menu of implementation strategies was used to increase the implementation outcomes of both *InCharge Health* and *HU Toolbox.* The “train and educate stakeholders strategy” (41, 42) included ongoing consultation and training of patients and providers on how to use both interventions, both as one-on-one activities (patient or provider individual meetings while in clinic) and group activities (provider educational meetings during staff and faculty meetings). “Support of the clinicians”” strategy (41, 42) was utilized during the study as reminders to clinicians to use the HU Toolbox and the facilitation of relay of clinical data as a function of the *HU Toolbox* app (i.e., guidance on correct prescribing of hydroxyurea app function). Other implementation strategies included understanding barriers and facilitators to digital health interventions, a thorough understanding of the implementation context, and patient feedback. These strategies were prospectively tracked and documented by each participating site.

### Activity 4: Selection of implementation models and frameworks

Our challenge in organizing patient, provider, and clinic-level contextual factors within SCDIC included the selection of implementation frameworks that would appropriately represent the multiple dimensions of the intervention (two target levels and several mHealth features) while accounting for the complex context where the population received care and where we sought to enhance the impact of our intervention. For instance, unmet social needs can lead to health disparities (43) and may significantly influence how patients with SCD engage with the intervention. Recruitment efforts that lead to equitable reach across the patient population and equity in the adoption and delivery of the intervention among providers are of paramount importance in the SCD population, given that the majority belong to underserved groups in the United States. Additionally, SCD characteristics (e.g., the disease severity) and co-morbidities might also affect response to the intervention (i.e., response heterogeneity) and lead to a lack of robust effects. Given the variability in patient characteristics across the participating sites, this was of particular concern.

SCDIC investigators considered several models and frameworks to define theory-based domains associated with contextual variables and the overall robustness of the mHealth innovation. Our goal was to examine the influences of patient, provider, and clinical setting level characteristics on study outcomes and the qualitative examination of barriers and enablers of the mHealth innovation. Given that different models and frameworks identify other metrics for evaluating implementation success, the group decided to combine the Consolidated Framework for Implementation Research (CFIR) (44) and the Reach Effectiveness Adoption Implementation and Maintenance (RE-AIM) (45) frameworks. CFIR is a determinant framework with five domains (inner setting, outer setting, intervention, process, and individuals involved) that systematically assesses potential barriers and facilitators (the determinants) of an implementation. RE-AIM is a versatile planning and evaluation framework, in which dimensions (reach effectiveness, maintenance, adoption, and implementation) systematically capture the outcomes of the implementation while assessing the equitability of the implementation (46). Because we planned qualitative data collection as part of our formative evaluation of the implementation process, we used RE-AIM Qualitative Evaluation for Systematic Translation (RE-AIM QuEST) to develop the interview questions (47).

In addition to CFIR and RE-AIM/RE-AIM QuEST, we also used the Technology Acceptance Model (TAM) (48, 49) to design and measure the outcomes specifically associated with mHealth use. Users’ acceptance of new technology, including new mHealth innovations, impacts its successful adoption. TAM is a conceptual model that explains the intent to use new information technology (including mHealth) or information science among users, including medical providers. TAM has five constructs: perceived usefulness, perceived ease of use, compatibility, mobile health care systems self-efficacy, and technical support and training. However, perceived ease of use and perceived usefulness are the two dominant determinants of technology use. mHealth care systems self-efficacy is the health care professional's perception of their ability to use health care systems to accomplish the health care task and must be accounted for when new technology is implemented. The combination of implementation frameworks RE-AIM/RE-AIM QuEST, CFIR, and TAM informed the planning of the study while complementing each other in evaluating the complexity of the influential multi-level factors on implementation (Figure 2). TAM was used to measure the specific constructs related to the intervention/m-Health tool. CFIR was used in specific quantitative measures of intervention characteristics, characteristics of individuals, inner settings, and processes. The five RE-AIM domains (Reach, Effectiveness, Adoption, Implementation, Maintenance) were used to evaluate implementation outcomes with quantitative and qualitative assessments.

### Activity 5: Evaluation plan

To visualize the potential interrelatedness of the multiple influential factors of the context, we created a matrix that grouped CFIR and TAM domains and mapped them to all 5 RE-AIM domains for both the patient-level (Supplementary Table S1) and provider-level (Supplementary Table S2) strategies. We designed specific plans to examine how influential factors of app utilization potentially moderate the relationship between (1) how the level of *InCharge Health* implementation might correlate with hydroxyurea adherence and (2) how the level of *HU Toolbox* implementation might correlate to increases in providers' knowledge and self-efficacy in prescribing hydroxyurea. To assess outcomes within each RE-AIM domain, quantitative measures were selected with possible moderation or mediation by CFIR constructs (Supplementary Tables S1, S2). In mixed methods evaluation, qualitative data complements and expands, within key themes, the “*why*” and “*how”* barriers and facilitators to recruitment, implementation, and sustainability affect implementation. The description of all measures utilized in this study and their frequency have been previously described (17). In brief, quantitative surveys are given at baseline and study exit, while qualitative data (semi-structured interviews) are conducted at the end of study participants at each site.

## Discussion

Dissemination and Implementation research has a growing number of models and frameworks, and parsimoniously using them is essential, but this is not always possible when there is high complexity in contextual factors and intervention components. While there might exist a need to combine aspects of various theories, models, and frameworks in designing interventions and complex evaluations, the methods for approaching this blending process are not widely available. Our study serves as a case study demonstrating the process of co-developing a multi-level/multi-component intervention and designing its evaluation. In our study, we combined HBM, SCT, RE-AIM, CFIR, and TAM while incorporating the broad perspectives of the diverse team members to plan and operationalize the frameworks, measurements, and evaluation. This manuscript outlines a rationale, process, and application that may be useful as an example for others to consider in designing an implementation evaluation, particularly around electronic health interventions to improve medication adherence in chronic diseases.

When clinical guidelines are implemented, theories, models, and frameworks are not always used to guide intervention and strategy development or their evaluation (50, 51). When theories are not used to plan, undertake, or evaluate implementation, the correct diagnosis of the underlying reasons for the success or failure of implementation (i.e., the determinants or the barriers and facilitators of low guideline adoption) may not be identified, therefore reducing the likelihood of effective interventions. Our study exemplifies how theories, models, and frameworks can be used to guide the entire process of planning, execution, and evaluation of guideline implementation, in our case, the utilization of hydroxyurea in SCD.

The process evaluation of a multi-level implementation study is complex and includes considerations of potential interactions between the intervention elements and the targeted levels of the intervention. Additionally, in chronic diseases, where there is substantial variability among the clinical characteristics of the patient, patient and organizational care settings, stakeholder perspectives, and the health care provider's expertise level, attention to contextual factors is particularly relevant when interpreting the effects of the intervention on outcomes. The careful development of an evaluation process that accounts for the different intervention and contextual components is thus paramount but potentially very complex. The description of the development of the process evaluation of a multi-faceted intervention can advance our understanding of their effects by illustrating the evaluation process design.

### Lessons learned

Over the course of 19 meetings, a 16-member multidisciplinary implementation team within the SCDIC conceived and implemented a project that utilized Intervention Mapping to guide the process, which leveraged and combined different models and frameworks to plan the evaluation of the implementation of a multi-level intervention to increase adherence to the evidence-based therapy for individuals with SCD, hydroxyurea. Our team tackled the difficult task of addressing a vital problem in the SCD field (the low hydroxyurea uptake) by overcoming the challenges of forming a functional and integrated diverse team that had first to learn how to work together, share knowledge, and develop the conceptual models, to create the interventions, select, and combine implementation frameworks, and design the evaluation plans that are appropriate for use in complex multilevel interventions. Our diverse community of scientists, government representatives, clinicians, and patient partners recognized that a proper understanding of the implementation process would require careful consideration of the multi-level factors that can influence the implementation in the SCD population. Embedded in this concept was the notion that for the group to properly function and advance the research question, developing trust, respect, and having a shared vision was essential. The research team integration followed collaboration and team science principles, which set clear expectations for sharing credit, authorship, and maintaining self-awareness (52). Strong communication was at the core of our team's functioning. The group consensus was initially slow to develop in trying to choose the design of the intervention (i.e., targets, function) and which model or framework for evaluation. Implementation researchers came with knowledge but often loyalty to a particular model, while clinical investigators were new to implementation methods and evaluation. The process creating a functional partnership between implementation science researchers and clinical investigators underscored the need to develop a “common language” between both teams while investing time to build knowledge of the respective fields across all team members. Organizing our thinking around a logic model and focusing on the “diagnosis of the implementation gap” (i.e., the determinants of the hydroxyurea utilization gap (illustrated in Figure 1) and choosing the frameworks second was instrumental in creating efficiency. Fortunately, there was sufficient lead time and support from the funding agency to examine the strengths and drawbacks associated with various models and frameworks and the possibility of combining aspects of each to reach an agreement. Accounting for the lead time (and possible delays) in creating multidisciplinary research teams and reaching team integration is important and should not be overlooked.

Our selection of theories, models, and frameworks followed our needs assessment phase. For this project, no models were adapted to fit the study intervention or evaluations. However, for some frameworks, not all constructs were applicable. For instance, the “Mobile health care systems self-efficacy” driver within TAM was less pertinent to developing the provider-level intervention. That's because, among SCD providers, the perception that mHealth could help with the task of prescribing hydroxyurea was less of a factor when the dominant perceived ease of use and usefulness of the intervention were used (i.e., judging if mHealth was helpful was less important than how much this tool could increase their productivity). Additionally, the outer setting construct within CFIR was not examined, as implementing mHealth in clinics is mainly controlled by the local leadership and policies and generally not regulated by the existing health systems policies. We did not encounter problems aligning our outcomes and influential contextual factors to the existing domains and constructs of the frameworks used. However, the issue of problems aligning with existing domains may occur depending on the research question, and population studied. Therefore, the careful selection of models and frameworks deserves extra attention and effort from the investigators, as adaptations to existing models and frameworks may be required.

Our work describing the process development of how to combine implementation models and frameworks to study how multi-level contextual factors optimally and comprehensively affect implementation outcomes adds to this growing literature. When models and frameworks are combined to evaluate the implementation of an intervention or practice, essential factors that caused the organization to reject or accept the intervention can be uncovered. For instance, formally appointing key stakeholders to ensure the fidelity of the intervention or gaining visible support from the system and local leaders are contextual factors that are not necessarily known as pre-conditions for optimal implementation (53, 54). While CFIR has been used to evaluate the implementation of the transition to adult care activities in SCD (55), rare examples of the combination of different models and frameworks in SCD exist (56), and none focused on medication utilization. Whereas RE-AIM provides a practical framework for planning and evaluating mHealth interventions, other models such as TAM and CFIR could explain *why* implementation might succeed or fail if used proactively and help to identify relevant modifiable factors affecting adoption, implementation, and maintenance. By understanding the *why*, we hope to identify mediators and moderators of the intervention and narrow down the components of the intervention that should be modified, removed, or new ones that need to be created in further adaptation while shedding light on the mechanism of interventions and identification of new implementation strategies. Finally, adding qualitative data to CFIR will allow us to map the level of influence on the CFIR constructs and domains. A qualitative design can expand quantitative data and provide new hypotheses to explain *why* implementation succeeds. Therefore, adding a qualitative design when using CFIR can be useful.

### Limitations

Our process to select the theories, models, and frameworks was iterative and followed matching theories and strategies to the type of intervention we sought to implement to best fit the quality gap we were trying to address, namely, low hydroxyurea utilization in SCD. Our process ensured diverse perspectives and consensus. It is possible, however, that this selection was not optimal. For example, our choice of models and frameworks preceded the publication of the RE-AIM expansion, which recognizably could represent an alternative to our approach. RE-AIM and PRISM can also be combined to investigate contextual predictors of the implementation outcomes, and an expanded version of RE-AIM has recently been published (57). Although the process development of the intervention and its evaluation were carefully developed and reported, not all implementation strategies might have been pre-identified for tracking. This may limit our ability to evaluate our implementation in the future fully. A pre-specification and comprehensive identification of all implementation strategies need to be done, and appropriate time should be allotted to this activity during study planning.

## Conclusions

In conclusion, we report the processes of developing a multi-level, multi-component intervention to foster greater use of hydroxyurea therapy among patients with SCD and detail this multi-level intervention and our comprehensive plan for its evaluation. Careful consideration of how the multiple components of the intervention can interact with the various targets and contextual factors will facilitate the description of the implementation's how, when, what, where, and who of the implementation and the *why*. The results of our mHealth adherence-enhancing study and future research will refine the evaluation approach to create new knowledge in developing an evaluation model for multi-level interventions to increase hydroxyurea uptake among adolescents and adults with SCD.

## Data Availability

The original contributions presented in the study are included in the article/**Supplementary Material**, further inquiries can be directed to the corresponding author/s.
