# Supplementary material for: Evaluating the implementation of a multi-level mHealth study to improve hydroxyurea utilization in sickle cell disease
Source: Front Health Serv. 2023 Jan 20;2:1024541. doi: 10.3389/frhs.2022.1024541 (PMC10012741; doi:10.3389/frhs.2022.1024541)
Supplement: Supplementary file 1 [file Table1.docx]

| **Supplemental Table 1. Matrix of Frameworks for patient-level intervention (*InCharge Health* app)** | | | | | |
| --- | --- | --- | --- | --- | --- |
| **RE-AIM Domain** | | **TAM Domain** | **CFIR Domain** | **CFIR Construct** | **Measurement tools and data sources** |
| Reach (characteristics that influence motivation to accept or reject the *InCharge Health* intervention) | - Sociodemographic characteristics of patients at each site   ‎   - Proportion and representativeness of patients screened for the study (numerator) among all patients who receive hydroxyurea treatment (denominator) at each site   ‎   - Proportion and representativeness of patients eligible for the study (numerator) among all patients who receive hydroxyurea treatment (denominator) at each site   ‎   - Proportion and representativeness of patients participating/enrolled in the study (numerator) among all patients who receive hydroxyurea treatment and were eligible (denominator) at each site |  | Characteristics of individuals | Knowledge & beliefs about the intervention | - Perceived usability and acceptability of mHealth intervention scale(1) - Qualitative interview of patients - Prescription drug filling data |
|  |  |  |  | Self-efficacy | - Patient quantitative data collection form for self-efficacy - Qualitative interviews of patients |
|  |  |  |  | Other personal attributes:  Disease severity  Duration of HU treatment prior to study  Prior biological response to HU  Pain interference  Health literacy  Self-reported HU adherence  Perceived past experiences with HU | - Chart review for laboratory markers and utilization data - ASCQ-me and PROMIS quality of life measures   Medical chart abstraction:   - frequency of SCD complications, - date start HU, - other medications, and - fetal hemoglobin level - Patient quantitative data collection form for perceived adherence - Qualitative interviews of patients |
| Effectiveness (characteristics that influence clinical effect of the *InCharge Health* intervention) | - Change in hydroxyurea adherence among those receiving the intervention   Change in Quality of life, self-efficacy  ‎   - Change in Emergency department visits, hospitalizations - Change in laboratory markers of hydroxyurea effect   ‎ | Perceived usefulness  Perceived ease of use | Characteristics of individuals | Knowledge & beliefs about the intervention | - Perceived usability and acceptability of mHealth intervention scale(1) - Qualitative interview of patients - Prescription drug filling data |
|  |  |  |  | Self-efficacy | - Patient quantitative data collection form for self-efficacy - Qualitative interviews of patients |
|  |  |  |  | Other personal attributes:  Disease severity  Duration of HU treatment prior to study  Prior biological response to HU  Pain interference  Health literacy  Self-reported HU adherence  Perceived past experiences with HU | - Chart review for laboratory markers and utilization data - ASCQ-me and PROMIS quality of life measures   Medical chart abstraction:   - frequency of SCD complications, - date start HU, - other medications, and - fetal hemoglobin level - Patient quantitative data collection form for perceived adherence   Qualitative interviews of patients |
| Adoption (characteristics that influence organization’s motivation to accept or reject the *InCharge Health* intervention) | - Proportion and description of clinics in each site agreeing to support *InCharge Health* - Proportion and description of providers in each clinic agreeing to support *InCharge Health* (ie, proportion enrolled on the study) | Compatibility  Technical support and training | Intervention characteristics | Adaptability  Intervention source | - Perceived usability and acceptability of mHealth intervention scale(1) - Clinic administrative data and data collection forms   ‎   - Qualitative interviews with patients, providers, and administrators - Organizational readiness survey   ‎ |
|  |  |  | Inner setting | Leadership engagement  Culture  Implementation climate  Compatibility  Relative priority  Readiness for implementation  Leadership engagement  Available resources  Access to knowledge & information |  |
| Implementation (consistency of delivery of the *InCharge Health* intervention) | - Consistency with which sites can implement the app as planned   ‎   - Qualitative assessment of any adaptations or enhancement to recruitment strategies needed to meet enrollment by the clinic, by site   ‎   - Assess adaptation of training needed to improve *InCharge Health* implementation at each site   ‎   - Engagement with the app: percentage, number, and representativeness of patients who used *InCharge Health* during the study period (low, medium-low, medium, or high use; in the entire practice)   ‎   - Proportion, number, and characteristics of patients who complete the study among those who initiate the use of the app but then later discontinue at each site   ‎   - Percentage and characteristics of patients who reported satisfaction with the *InCharge Health* app   ‎   - Clinic/provider assessment of perceptions of *InCharge Health* app for further scale-up or sustainability—ease of use, preferred features |  | Intervention characteristics | Relative Advantage  Adaptability  Complexity | - App usage statistics   ‎   - Qualitative interviews with patients, providers, and administrators - Perceived usability and acceptability of mHealth intervention scale(1) - Clinic data collection forms |
| Maintenance/sustainability (extent that the *InCharge Health* intervention is routinely used for patient care) | - Extent to which program leaders express a desire or intent to continue providing the app with patients at the conclusion of the research   ‎   - Percentage of patients who continue to use the app beyond the study period and their representativeness   ‎ |  | Process | Engaging  Opinion leaders  Champions  Reflecting and evaluating | - Pharmacy claims data   ‎   - App use statistics   ‎   - Clinic data collection forms   ‎   - Qualitative interviews of administrators and providers - Organizational readiness for change survey‎ |

Note: 1. Stoyanov SR, Hides L, Kavanagh DJ, Zelenko O, Tjondronegoro D, Mani M. Mobile app rating scale: a new tool for assessing the quality of health mobile apps. JMIR Mhealth Uhealth. 2015;3(1):e27.
