# Supplementary material for: Evaluating the implementation of a multi-level mHealth study to improve hydroxyurea utilization in sickle cell disease
Source: Front Health Serv. 2023 Jan 20;2:1024541. doi: 10.3389/frhs.2022.1024541 (PMC10012741; doi:10.3389/frhs.2022.1024541)
Supplement: Supplementary file 2 [file Table2.docx]

| **Supplemental Table 2. Matrix of Frameworks for provider-level intervention (*HU Toolbox* app)** | | | | | |
| --- | --- | --- | --- | --- | --- |
| **RE-AIM domains** | | **TAM Domain** | **CFIR Domain** | **CFIR Construct** | **Data sources** |
| Adoption—clinic (characteristics that influence organization’s motivation to accept or reject the *HU Toolbox* intervention) | Proportion and representativeness of clinics that agree to support the *HU Toolbox*  ‎ |  | Inner setting | Culture  Implementation climate  Compatibility  Relative priority  Readiness for implementation  Leadership engagement  Available resources  Access to knowledge & information | Institutional data to describe clinics (e.g., size, case mix, years in service, regional sociodemographic of SCD patients)  ‎  Clinic data collection form  ‎  Qualitative interviews of clinic administrators  Organizational readiness survey‎ |
| Adoption—provider (characteristics that influence providers’ motivation to accept or reject the *HU Toolbox* intervention) | Characteristics of providers at each site (e.g., specialty, years in practice, sociodemographic, level of expertise)  ‎  Proportion and representativeness of eligible providers approached in the study (numerator) among all providers (denominator)  Proportion and representativeness of enrolled providers in the study (numerator) among all eligible providers (denominator) at each site | Perceived usefulness  Perceived ease of use  Compatibility  Technical support and training | Intervention characteristics | Evidence Strength & quality  Relative advantage  Trialability  Complexity | Provider quantitative data collection form  Perceived usability and acceptability of mHealth intervention scale(1)  ‎  Clinic data collection form  Qualitative interviews of providers |
|  |  |  | Characteristics of individuals | Knowledge & beliefs of intervention |  |
| Effectiveness (characteristics that influence clinical practice effect of the *HU Toolbox* intervention) | Number and proportion of providers demonstrating improved knowledge and self-efficacy in hydroxyurea administration  ‎  Percentage of patients who were prescribed hydroxyurea per provider |  | Characteristics of individuals | Self-efficacy  Other personal attributes (level of SCD expertise, years in practice, medical specialization) | Provider quantitative data collection form  ‎  Medical chart abstraction  Qualitative interviews of providers ‎ |
| Implementation (consistency of delivery of the *HU Toolbox* intervention) | Consistency with which sites can implement the use of the Toolbox app as planned  ‎  Engagement with the app: Percentage, number, and representativeness of providers that appropriately used *HU Toolbox* app (low or high use; in the entire practice)  Percentage of providers who reported satisfaction with *HU Toolbox* app (Perceived usability and acceptability of mHealth intervention scale(1))  Percentage of patients whose provider used the Toolbox at each site‎ |  | Process | Planning  Engaging  Champions  Reflecting & evaluating | Clinic data collection form  App usage statistics  ‎  Qualitative interviews of providers ‎and clinic administrators‎ |
| Maintenance/sustainability (extent that the *HU Toolbox* intervention is routinely used for patient care) | Extent to which program leaders express a desire or intent to offer or encourage the use of the Toolbox app by their clinical providers at the conclusion of the research  ‎  Percentage of providers who continue to use the provider app beyond the study period, and representativeness  ‎  Percentage of providers who continue to prescribe hydroxyurea to their patients |  | Process | Planning  Engaging  Champions  Reflecting & evaluation | Clinic data collection form  App usage statistics  ‎  Qualitative interviews of providers ‎and clinic administrators‎ |

Note: 1. Stoyanov SR, Hides L, Kavanagh DJ, Zelenko O, Tjondronegoro D, Mani M. Mobile app rating scale: a new tool for assessing the quality of health mobile apps. JMIR Mhealth Uhealth. 2015;3(1):e27.
